# An In-Depth Examination of the Natural Radiation and Radioactive Dangers Associated with Regularly Used Medicinal Herbs

**DOI:** 10.3390/ijerph19138124

**Published:** 2022-07-01

**Authors:** Heba A. Saudi, Heba T. Abedelkader, Shams A. M. Issa, Hanan M. Diab, Gharam A. Alharshan, Mohamed A. M. Uosif, Ibrahim I. Bashter, Antoaneta Ene, M. El Ghazaly, Hesham M. H. Zakaly

**Affiliations:** 1 Physics Department, Faculty of Science, Al-Azhar University (Girls Branch), Nasr City, Cairo 11884, Egypt; heba_saudi@azhar.edu.eg; 2 Physics Department, Faculty of Science, Zagazig University, Zagazig 44511, Egypt; hebathabt@yahoo.com (H.T.A.); ibashter@gmail.com (I.I.B.); 3 Physics Department, Faculty of Science, Al-Azhar University, Assuit 71524, Egypt; shams_isa@yahoo.com (S.A.M.I.); mauosiff@ju.edu.sa (M.A.M.U.); 4 Department of Physics, Faculty of Science, University of Tabuk, Tabuk 47512, Saudi Arabia; 5 National Center for Nuclear Safety and Radiation Control, Atomic Energy Authority, Cairo 11787, Egypt; hnndiab@yahoo.co.uk; 6 Physics Department, College of Science, Princess Nourah Bint, Abdulrahman University, P.O. Box 84428, Riyadh 11671, Saudi Arabia; gaalharshan@gmail.com; 7 Physics Department, College of Science, Jouf University, P.O. Box 2014, Sakaka 72388, Saudi Arabia; 8 INPOLDE Research Center, Department of Chemistry, Physics and Environment, Faculty of Sciences and Environment, Dunarea de Jos University of Galati, 47 Domneasca Street, 800008 Galati, Romania; 9 Department of Physics, Faculty of Science, Zagazig University, Zagazig 44519, Egypt; ghazaly2000@yahoo.com; 10 Institute of Physics and Technology, Ural Federal University, 620002 Ekaterinburg, Russia

**Keywords:** natural radiation, radioactive risks, medical herb, high-purity germanium

## Abstract

The specific activity of U-238 and Th-232, as well as K-40 radionuclides, in twenty-nine investigated medicinal herbs used in Egypt has been measured using a high-purity germanium (HP Ge) detector. The measured values ranged from the BDL to 20.71 ± 1.52 with a mean of 7.25 ± 0.54 (Bq kg^−1^) for uranium-238, from the BDL to 29.35 ± 1.33 with a mean of 7.78 ± 0.633 (Bq kg^−1^) for thorium-232, and from 172 ± 5.85 to 1181.2 ± 25.5 with a mean of 471.4 ± 11.33 (Bq kg^−1^) for potassium-40. Individual herbs with the highest activity levels were found to be 20.71 ± 1.52 (Bq kg^−1^) for uranium-238 (H4, Thyme herb), 29.35 ± 1.33 (Bq kg^−1^) for thorium-232 (H20, Cinnamon), and 1181.2 ± 25.5 (Bq kg^−1^) for potassium-40 (H24, Worm-wood). (*AACED*) Ingestion-related effective doses over the course of a year of uranium-238 and thorium-232, as well as potassium-40 estimated from measured activity concentrations, are 0.002304 ± 0.00009 (minimum), 0.50869 ± 0.0002 (maximum), and 0.0373 ± 0.0004 (average)(mSv/yr). Radium equivalent activity (*R*aeq), annual gonadal dose equivalent (*AGDE*), absorbed gamma dose rate (*D_outdoor_*, *D_indoor_*), gamma representative level index (I), annual effective dose (*AED*total), external and internal hazard index (*H*_ex_, *H*_in_), and excess lifetime cancer risk were determined in medicinal plants (*ELCR*). The radiological hazards assessment revealed that the investigated plant species have natural radioactivity levels that are well within the internationally recommended limit. This is the first time that the natural radioactivity of therapeutic plants has been measured in Egypt. In addition, no artificial radionuclide (for example, 137Cs) was discovered in any of the samples. Therefore, the current findings are intended to serve as the foundation for establishing a standard safety and guideline for using these therapeutic plants in Egypt.

## 1. Introduction

Many plants have been employed for nutrition and medicine since the dawn of human history. The study of the concentration of radioactivity in plants in the environment is relevant to ecological and plant evolution under certain geochemical conditions and adaptation and provides information for environmental radioactivity monitoring [1,2].

Radionuclides from the ^238^U and ^232^Th family, as well as ^40^K, are terrestrial primordial radionuclides that formed in the earth’s crust and are natural sources of radioactivity in the environment [3].

Traditional medicine is defined by the World Health Organization as therapeutic techniques that have existed for hundreds of years before the establishment and spread of modern medicine and are still in use today [4].The environmental conditions could affect the properties and efficacy of medicinal herbs, and one of the most significant parameters that should be controlled is the level of natural and artificial radionuclides. According to the WHO guidelines for herbal medicines’ quality regulation, the health risk posed by the accidental contamination of herbal medicines by radionuclides depends not only on the specific radionuclide and the level of contamination but also on the dose and duration of use of the product consumed [5].

All over the world, medicinal herbs have been used for a long time [6]. A growing number of people are turning to herbal medicine to enhance their health in recent years because of their well-known pharmacological as well as therapeutic properties of many of them [7]. Seventy-five percent of the world’s population relies on herbs for basic health care, according to WHO reports [8]. We are witnessing a global herbal that is taking place all over the world, with herbs containing medicinal properties being used in contemporary medical therapies as well. A plant’s most used organs are its leaves. Other organs include the flowers, fruit, seeds, stems, wood, bark, roots, and rhizomes. These organs are used as is or pulverized into a fine powder [9]. Additionally, medicinal plant ethnobotanical research is a critical step in the local development of ecotourism, which includes environmental museums and small-scale businesses dealing with native medicinal and edible plants, as well as community-based bio-conservation initiatives. However, in order to complete all of these duties, the use of medicinal plants and their products must be strictly regulated in order to avoid any potentially harmful side effects on the health of consumers. Since plants are the principal conduit of natural radionuclides entering the human body through the food chain, radionuclides in soil may enter the food chain through direct deposition on leaves or transfer to portions of plants used for medicinal purposes. In addition, root uptake, direct deposition from the atmosphere, and resuspended natural radionuclide from the soil contribute to the absorption of soluble radionuclides in soil water. In soils and rocks, the naturally occurring radionuclides ^226^Ra, ^232^Th, and ^40^K are the principal radiation sources. Because of their gamma-ray emissions, these radionuclides constitute a danger of external exposure [10].

Medicinal herbs’ properties and efficacy may be influenced by their environmental surroundings, and one of the most important parameters that must be monitored is the level of natural and man-made radionuclides present in them. Aside from the specific radionuclide and the level of contamination, the health risks posed by accidental radionuclide contamination of herbal medicines has been found to be dependent on the amount consumed and the length of time it was consumed [5]. It is possible to accumulate harmful substances in the human body when using herbal remedies for a long period of time [11]. An individual’s annual effective dose from ingestion increases because of increased concentrations of radioactive elements, increasing the risk of radiological harm because of ingestion. As a result, it is critical to research radionuclide absorption and activity distribution, as well as the possible human effective radiation dosage from therapeutic plants. Medicinal plants can be found in their natural state or processed.

Due to preparation techniques that invariably eliminate part of the radionuclides, NORMS (Naturally Occurring Radioactive Material) activity concentrations in herbal formulations are substantially lower than in raw plants. The health effects of NORMS (Naturally Occurring Radioactive Material) exposure from medicinal plants and herbal preparation ingestion concerning NORMS levels in medicinal plants may be linked to most types of leukemia and cancer [12,13]. The average annual effective dose from natural sources is 2.4 mSv worldwide—the average radioactivity ingested in food and drink results in a dosage of roughly 0.29 mSv^−1^. Potassium, a vital nutrient, is the major radionuclide that contributes to the dosage. Potassium levels in the body are almost constant. Compared to uranium and thorium, thorium has a lower melting point [14,15]. K-potassium is the most important nutrients for plants. Because K and Cs are members of the same chemical element family, their attitude toward the plant’s metabolism is very similar to one another [16]. Potassium, as well as its naturally occurring radioisotope ^40^K, enters the plant roots through ion channels, or transporters, that are also used for the Cs^+^ ion transporter. As a result, a high K content in soil inhibits the adsorption of Cs, and the impact could be heightened by higher mobility of the potassium ion in soil, which increases the availability of potassium to plants [17]. The purpose of this study was to provide information on: natural radionuclide activity concentrations in numerous medicinal plants; the radiation hazards related to the intake of therapeutic plants, as evaluated in this research.

## 2. Materials and Methods

### Samples Preparation & Measurements

**Figure 1:**
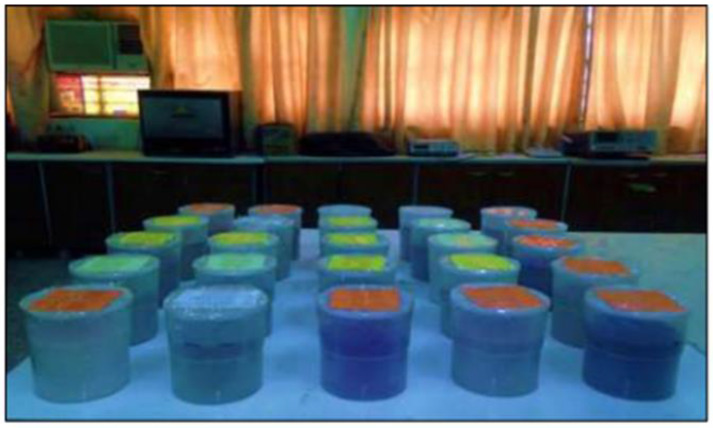
Samples inside the Marinelli beakers.

The Egyptian marketplaces provided dried medicinal plant samples measuring one kilogram apiece. The samples were then rinsed in water and dried in the sun to remove any dust contamination. At the central laboratory for Environmental Radioactivity Measurements, Intercomparison and Training CLERMIT and Nuclear & Radiological Regulatory Authority in Cairo, these samples were crushed into tiny bits, homogenized, and dried in an electric oven at 105–110 °C until they reached a consistent weight. The dry components were subsequently ground into a fine powder, and sieved at 0.5 mm in diameter, with a sealed joint in a beaker, as illustrated in Figure 1. Finally, the samples were kept at room temperature for about a month before counting, to allow the radionuclides ^226^Ra, ^222^Rn, and their daughters to approach earthly equilibrium.

**Figure 2:**
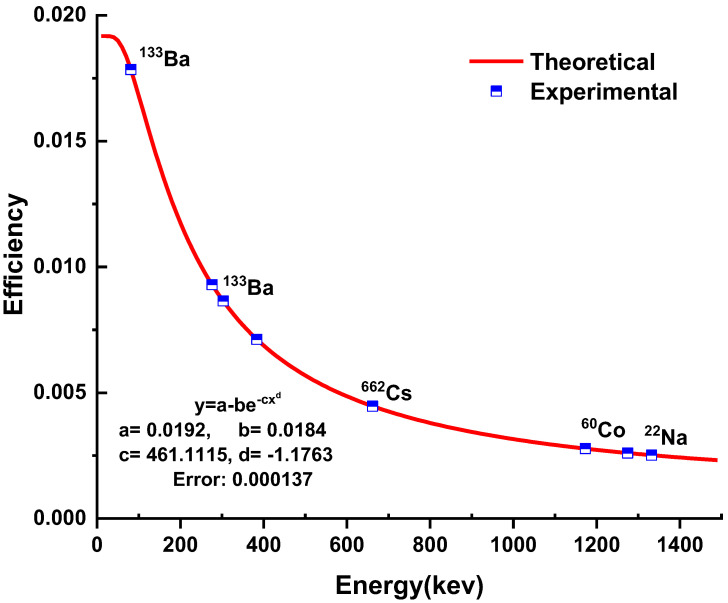
Standard sources are used to calibrate the detector for efficiency.

The dry mass of the samples in this experiment was used to calculate the radioactive content. Table 1 lists the traditional and scientific names. The samples have been counted using a gamma-ray spectrophotometer. A high purity germanium (HP Ge) detector with an efficiency of 25% and an energy resolution of 1.8 keV (FWHM) at a peak energy of 1333 keV from the ^60^Co, peak share to Compton 55:1 was used. Through an uninterrupted power supply, a high-voltage power supply (Model 13103) was used to deliver the bias voltage of 3000 V. (UPS). The detector was kept cooled in a 25-L Dewar with liquid nitrogen at 196 °C (77 K) and an ambient temperature of 16 to 27 °C. One-hundred millimeters of lead shielding reduces the soft components of cosmic rays to a shallow level. The X-ray (73.9 keV) generated by lead as a result of its interaction with external radiation was reduced by the copper layer [18]. To facilitate radionuclide identification and quantification, the system’s energy and efficiency were calibrated prior to the use of samples for analysis with the IAEA’s Multinuclear Reference Standard Solution, which has the same geometry of the investigsted samples as shown in Figure 2.

The standard and sample were computed for 8000 s to collect spectral data to improve counting and assessment. The activity concentrations of ^238^U, ^232^Th, and ^40^K, as well as the background in an empty beaker under the same conditions, were estimated after normalizing for background and heterogeneity [19]. The absolute efficiency was calculated using the next isotopes, which included (*E_γ_* and *I_γ_*%) ^133^Ba (80.1 KeV—34.06%) and (356 KeV—62.05%), ^137^Cs (661.6 KeV—85.12%), ^60^C (1173.2 KeV—99.97% and 1332.5 KeV—99.98%) and ^22^Na (1274.5 KeV—99.9%), with specified activities. The IAEA 154 instruction was used to calibrate the detector efficiency [20]. An equation fitted to experimental data by polynomial curve is reported in Formula 1. In this formula, Y is efficiency, a, b, c, d, e, f are constants, and x is the gamma ray energy in KeV.

Y = a + b (Lnx) + c (Lnx) ^2^ + d (Ln x) ^3^ + e (Lnx) ^4^ + f (Lnx) ^5^ (1)

The calibration curve fitted to experimental data by polynomial curve is shown in Figure 2. Background measurements, sample counting geometry, and a standard mixed source for efficiency calibration were all kept constant. All the spectra’s counting times were within 80,000 s. The absolute efficiency of detector arrangement was estimated using the registered gamma-ray spectrum:

(2) $$\varepsilon \left(E\gamma \right)=\frac{Net}{A\;x\;I\left(E\gamma \right)x\;T}\times 100\%$$

where the Net-area represents net counts for those that fall under the full-energy peak, A represents radionuclide activity at a given date, *I_γ_*(*E_γ_*) stands for the abundance of energy *E_γ_* and t represents counting. The radioactivity concentration of ^238^U, ^232^Th, and ^40^K in medicinal plants was assessed using quantitative analysis of the gamma spectra, acquired using Ortec MAESTRO-32 analytic software at specific energies. A mean of ^214^Pb (251.9 and 295.2 keV) and ^214^Bi (609.3 and 1764.5 keV) was used to compute ^238^U. A mean of ^208^Tl (2614.5 and 583.2 keV), ^212^Pb (238.6 keV), ^228^Ac(11.2 keV), and ^40^K(1460.0 keV) was used to calculate ^232^Th. After the decay had been corrected, the values for activity concentrations in decay chains were based on secular equilibrium for the various isotope activities. The measurement yielded no artificial radioactivity. Each sample’s radioactivity was determined using a calibrated high purity germanium detector. The radionuclides i in the samples had their specific activity (Asp (*E*, *i*) in Bq kg^−1^) evaluated using the following equation [21].

(3) $$asp\;\left(E,\;i\right)=\frac{Nsam\;\left(E,\;i\right)}{\epsilon_{\gamma }\;\left(E\right)\;T_{C\;}P_{\gamma }\;\left(E,\;i\right)Msam}\;$$

*Nsam*(*E*, *i*) is net counts under the full-energy peak corresponding to the *Ei* energy, *T_c_* is the calculation of live time (s), *P*_γ_(*E*, *i*) is the gamma emission potential of the radionuclide i to transition at energy *E*; *Nsam* is the dry weight of the samples (kg) after obtaining the values of the specific activity concentrations of radionuclides that occur naturally in medicinal plants. *ϵ*_γ_(*E*) is the absolute efficiency of detector. The equations used to calculate the radiological hazards have been discussed in detail in our previous works [22,23,24,25,26,27,28,29,30]. The error associated with every calculation was measured by the standard deviation (SD) equation. The disintegration of a radionuclide is a random process, and only an estimate of the true activity of a sample can be obtained. Factors such as the confidence limit and sample counting error are all dependent on counting time. When many samples with low-level activities must be assessed, it is important to utilize the time available in the most efficient manner. The percentage of sample counting error for the radioactivity measurement is found with the help of the following relation [31]:

(4) $$\sigma =\sqrt{\frac{N_{t}}{T^{2}{}_{t}}+\frac{N_{b}}{T^{2}{}_{b}}\;}$$

where *σ* is the standard deviation; *N_t_* is the number of counts for samples; *N_b_* is the counts for the background; *T_t_* is the counting time for *N_t_*, and *T_b_* is the counting time for *N_b_.*

## 3. Calculation of Radiological Hazard

The air-absorbed dose rate ($D_{out}$) was determined using UNSCEAR’s recommendations. The absorbed gamma dose rate *D* (nGy/h) in the air at 1 m above the ground was measured to guarantee the homogeneous dispersion of radionuclides. This parameter may be used to measure any radiological risk and radiation exposure from radionuclides in the soil; the absorbed dose rate in air *D_out_* was determined using the formula [32]:

(5) $$D_{out}=\frac{427}{1000}\times C_{Ra}+\frac{623}{1000}\times C_{Th}+\frac{43}{1000}\times C_{K}\;$$

where $D_{out}$ is the dose rate in nGy h^−1^ and $C_{Ra}$, $C_{Th}$, and $C_{K}$ are the activity concentrations (Bq kg^−1^) of radium (^226^Ra), thorium (^232^Th), and potassium (^40^K), respectively. Determining the ratio of the absorbed dose to the outdoor dose received from radiation emitted by radionuclides is a key step in health risk assessments.

The internally absorbed gamma dos (*D_in_*) rate is expressed by Equation (6), and according to the UNSCEAR 2000 report, this internal dose should not exceed 84 nGy/h [32].

(6) $$D_{in}=\frac{92}{100}\times C_{Ra}+\frac{110}{100}\times C_{Th}+\frac{8.1}{100}\times C_{K}\;$$

The average annual committed effective dose (*AACED*) for the ingestion of NORMs in medicinal plants is calculated using the expression:

(7) $$E_{AV}=C_{r}\times DCF_{ing}\times A_{i}\;$$

where $E_{AV}$ is the average annual committed effective dose, $C_{r}$ is the rate of consumption of intake NORMs from medicinal plants, $DCF_{ing}$ is the dose conversion coefficient for ingestion for each radionuclide (i.e., 4.5 × 10^−5^ 2.8 × 10^−4^, 2.3 × 10^−4^ and 6.2 × 10^−6^ mSv/Bq for ^238^U, ^226^Ra, ^232^Th, and, K respectively for an adult) [33], and $A_{i}$ isthe specific activity concentration of each radionuclide. Although there is no defined dosage for the use of medicinal plants, a rise in the rate of intake by a patient who utilizes these plants to treat an illness on a regular basis raises their average effective yearly dose Using the Formula (7), The average bound annual effective dosage for NORMs in medicinal plants is $E_{AV}=0.3\;\mathrm{mSv}/\mathrm{yr}$ [33]. $C_{r}$ represents the annual consumption rate of NORMs in medicinal plants, which is 1.8 kg/yr [1,2].

For all the medicinal plants utilized in this investigation, it was assumed that a patient requires 100 mL/day of the herbal preparation or product throughout the treatment period or is 5%.

To assess the health effects of the absorbed dose, the annual effective dose should be calculated using a conversion factor (0.7 mSv/yr) to convert the air-absorbed dose to the effective dose received by humans, along with an external occupancy factor (0.2), which is equivalent to a 20% outdoor occupancy and an 80% inward occupancy [34,35].

This variable is appropriate for identifying the lifestyle in the research area [36]. It can be used to compute the annual effective dose rate (*AEDR*, in mSv/y) received by a population. This component is appropriate for identifying the life pattern in the research area. The population’s annual effective dose rate (*AEDR*, in mSv/yr) may be computed using the following equation [37].

(8) $$AEDR_{out}=D_{out}\left[\frac{nGy}{h}\right]\times 8760\left[\frac{h}{yr}\right]\times 0.7\;\left[\frac{Sv}{Gy}\right]\times 103\;\left[\frac{mSv}{10^{-9}}\right]\times 0.2\;=D\times 1.2264\times 10^{-3}\left[\frac{mSv}{yr}\right]$$

where, $D\left[\frac{nGy}{h}\right]$ is the total air absorbed dose rate in the outdoors; 8760 h is the number of hours in one year; 0.2 is the outdoor occupancy factor; 0.7 $\frac{Sv}{Gy}$ is the conversion coefficient from the absorbed dose in the air to the effective dose received by adults; $10^{-6}$ is the conversion factor between nano- and milli-level measurements. The annual effective dose rates (E) are an important parameter to consider when evaluating the health effects of an absorbed dose. The conversion coefficient from absorbed dose in the air to effective dose (0.7 Sv/Gy) and the indoor occupancy factor (0.80) proposed by [13,32] are used to estimate effective dose rates. The annual effective dose in millisieverts per year (mSv/y) was calculated using the following formula [32].

(9) $$E_{in}=D_{in}\left[\frac{nGy}{h}\right]\times 8760\left[\frac{h}{yr}\right]\times 0.7\;\left[\frac{Sv}{Gy}\right]\times 0.8\times 10^{-6}\;\;=D_{in}\times 4.9056\times 10^{-3}\left[\frac{mSv}{yr}\right]$$

The thyroid gland, lungs, bone marrow, gonads, and breasts are among the organs affected by atomic radiation. The amount of *AGDE* produced in soil by the activity of ^226^Ra, ^323^Th and ^40^K is calculated as follows [32].

(10) $$AGDE\;\left(\mu {\mathrm{Sv}\;\mathrm{yr}}^{-1}\right)=\frac{309}{100}\times C_{Ra}+\frac{418}{100}\times C_{Th}+\frac{314}{1000}\times C_{K}$$

The external hazard index (*H*_ex_) produced by the emitted rays of the samples should be ≤1, which corresponds to the upper limit of *R_aeq_* (370 Bp/Kg) [38]. The *H*_ex_ external hazard index, expressed in (mGy/yr) is calculated according to the following equation

(11) $$H_{\mathrm{ex}}=\frac{1}{370}\times C_{Ra}+\frac{1}{259}\times C_{Th}+\frac{1}{4810}\times C_{K}\leq 1$$

where $C_{Ra}$, $C_{Th}$, and $C_{K}$ are the activity concentrations of ^226^Ra, (^238^U-series), ^232^Th, and ^40^K, respectively. The internal exposure *H*_in_ to ^222^Rn and its daughter products are controlled by an internal hazard index *H*_in_, which is defined in [39,40].

(12) $$H_{\mathrm{in}}=\frac{1}{185}\times C_{Ra}+\frac{1}{259}\times C_{Th}+\frac{1}{4810}\times C_{K}\leq 1\;$$

The radioactivity level index used to estimate the level of gamma radiation hazard associated with different concentrations of some specific radionuclides is defined by the following equation [21,41,42].

(13) $$I_{\gamma }=\frac{1}{150}\times C_{Ra}+\frac{1}{100}\times C_{Th}+\frac{1}{1500}\times C_{K}$$

where, $C_{Ra}$, $C_{Th}$, and $C_{K}$ are the activity concentrations of ^226^Ra, (^238^U-series), ^232^Th, and ^40^K, respectively. Even in the absence of radioactive components, miners, and inhabitants of the study region who are expected to spend the majority of their time in this environment, one may estimate carcinogenic potential using the lifetime cancer risk (*ELCR*). Excess lifetime cancer risk (*ELCR*) was determined based on the values of the annual committed effective dose using the equation

(14) ELCR=AACED×Average duration of life [DL]×Risk factor [RF]

where LE is life expectancy taken to be 70 years and RF is a fatal risk factor per sievert which was 0.05 [43].

## 4. Results and Discussion

**Figure 3:**
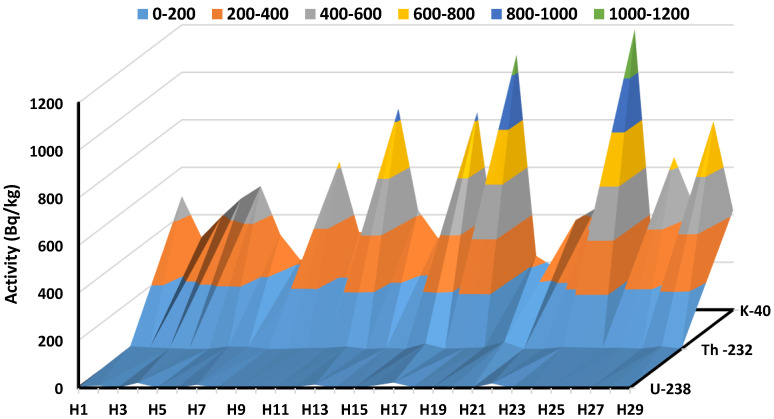
The activity concentration for ^238^U, ^232^Th, and ^40^K in medicinal plant samples (Bq⁄kg).

Gamma-ray spectrometry was used to measure the radioactivity levels of NORMs in 29 different medicinal plants that are commonly used in Egypt. The equation used to figure out the average concentrations of ^226^Ra, ^232^Th and ^40^K that were used (3). Calculations were also used to figure out how much radiation these medicinal plants might cause. The risk indexes and annual effective doses were also considered. Results from our study were compared to global averages set by UNSCEAR and results from other countries. Our findings and comparisons are shown in the following logical order. Figure 3 and Table 2 show the average dry weight activity concentrations of ^226^Ra, ^232^Th and ^40^K for the medicinal plants that were tested in this study. Each sample and isotope being looked at has a wide range of activities. Different medicinal plants may have different concentrations of NORMs because they have different amounts of radioactive minerals and can absorb certain elements [2].

From the current research, we can see that the concentration levels of ^238^U varied from BDL to 20.71 ± 1.52 Bq/Kg as observed in 15 plant species exceeding BDL values with an average of 7.25 ± 0.52 Bq/Kg. Thyme herb (H4) has the highest ^238^U concentration. ^232^Th concentrations ranged from BDL to 29.35 ± 1.33 Bq/Kg, as observed in 10 plant species with with concentrations greater than BDL values, with an average of 7.78 ± 0.633 Bq/Kg. Cinnamon (H20) has the highest ^232^Th concentration. The ^40^K activity concentrations were recorded between 172 ± 5.85 Bq/Kg turmeric and 1181.2 ± 25.5 Bq/Kg cinnamon with an average value of 471.4 ± 11.33 Bq/Kg. Since some of the studied samples have been imported from different regions, the detected activity values of radionuclides were affected due to different levels of natural radioactivity in the soil and environment in those countries. Nevertheless, based on the findings, the specific activity values of ^238^U were within the limit of 33 Bq/Kg in all samples [32].

**Figure 4:**
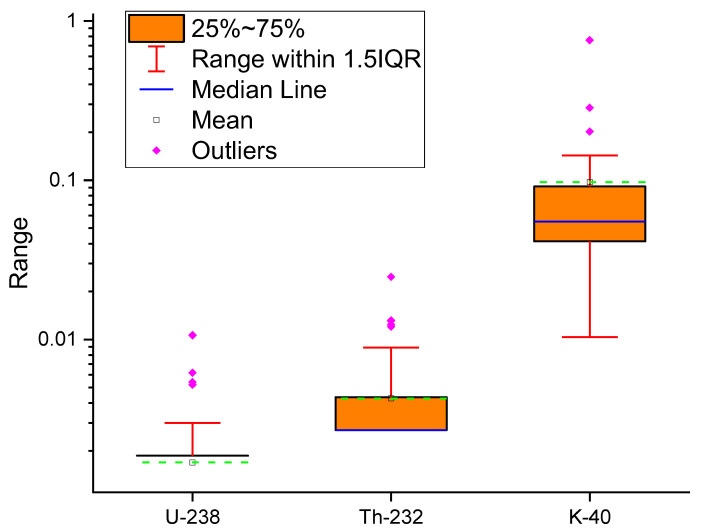
Range, mean, median line, and outlier radioactive elements (^238^U, ^226^Ra, ^232^Th, and ^40^K radionuclides) for measured samples in the interested area.

**Figure 5:**
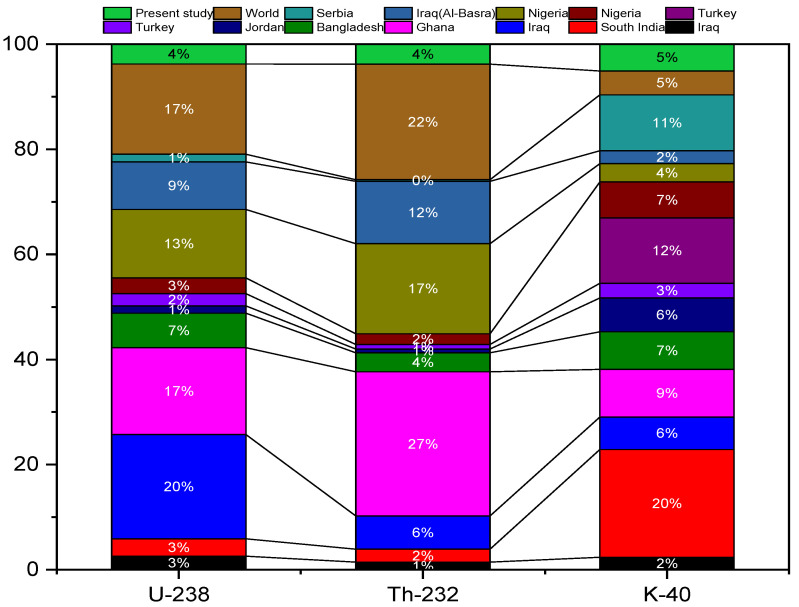
The mean activity concentrations of natural radioactivity of medicinal plant samples in the present study were compared with those from similar investigations performed in other countries.

Furthermore, it was discovered that the specific activity levels of ^232^Th in all samples were within the range of 45 Bq/Kg [32]. Except for a few samples that were more extensive than the permissible value of 400 Bqkg^−1^ [32], the values of the activity concentration of the ^40^K are less than the allowable value of 400 Bqkg^−1^. Since typical radionuclide activity heights are not regulated across the ground and due to the flowers’ ability to absorb more basic features than others, differences in the concentrations of activity could be attributed to changes in the physical location of the plants and the radiochemical action of the lands in which these medicinal plants are developed or cultivated. The increased potassium activity in these plants might be related to the plants’ effectiveness in absorbing potassium as well as other components from the soil [44]. Figure 4 shows the range, mean, median line, and outliers’ radioactive elements for measured samples in the region of interest. The current study’s activity concentration findings were compared to the published data in Table 3 for a selection of medicinal plants found in the literature, as shown in Figure 5. This comparison shows that the current findings are relatively consistent with those measured in other nations using the global values indicated in the UNSCEAR 2000 report.

In a real sense, the current results show that the amount of ^238^U in the air is much higher than in Iraq [45], South India [46], Jordan [49], Turkey [50], Nigeria [53], Serbia [54], Turkey [51], and lower than amounts obtained in Iraq [47], Ghana [2], Bangladesh [48], and Nigeria [53]. The results of ^232^Th show that our result is higher than the results found in Iraq [45], South India [46], Nigeria [52], Jordan [49] and Turkey [50] and is lower than the results found in Iraq [47], Ghana [2] and Nigeria [53]. Our findings in the case of ^40^K are significantly greater than those from Iraq [45], Turkey [50] and Nigeria [53] but significantly lower than those from Iraq [47], South India [46], Ghana [2], Jordan [49], Nigeria [52] and Turkey [51].The discrepancies in natural radioactivity concentrations between countries might be explained by the raw material sources (Figure 5).

**Figure 6:**
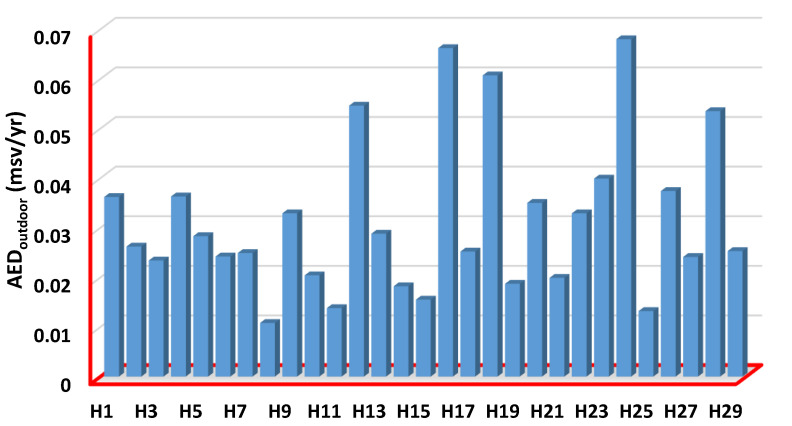
Outdoor annual effective doses (*AED_outdoor_*) for all herbs.

**Figure 7:**
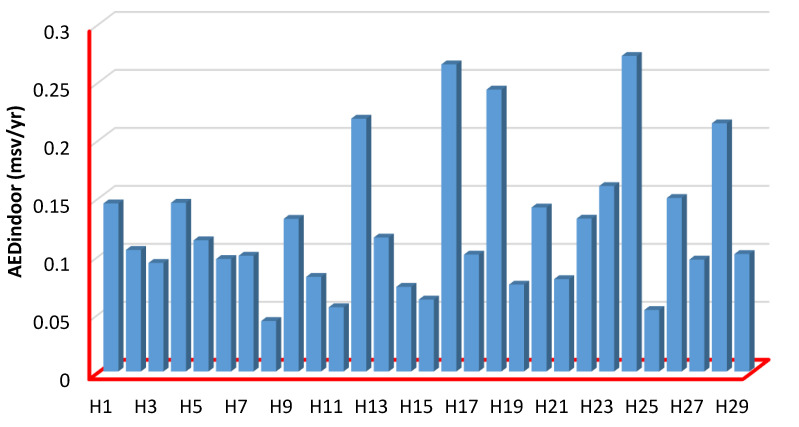
Indoor annual effective doses (*AED_indoor_*) for all herbs.

The measured outdoor annual effective doses (*AED_outdoor_*) values for examined herbs have been listed in Table 4. The values ranged from 0.0108 ± 0.0032 to 0.0680 ± 0.0097 mSv/yr, with the mean value of 0.0315 ± 0.0084 mSv/yr. Lemon Balm (H8) and Worm Wood (H24) herbs have the lowest and highest *AED_outdoor_* among all herb samples (Figure 6). The *AED_outdoor_* results are smaller than the corresponding global value of 1 mSv/yr. The measured indoor annual effective doses (*AED_indoor_*) values for examined herbs have been listed in Table 4**.** The values ranged from 0.0810 ± 0.0032 to 0.5053 ± 0.0097 mSv/yr, with the mean value of 0.236 ± 0.0084 mSv/yr. Lemon Balm (H8) and Quince (H23) herbs have the lowest and highest *AED_indoor_* and *AED_outdoor_* among all herb samples (Figure 6 and Figure 7). The *AED_indoor_* results are smaller than the corresponding global value of 1 mSv/yr. 0.0919 ± 0.0036, 0.555 ± 0.024, and 0.267 ± 0.0095 mSv/yr are the minimum, maximum, and average total annual effective dose (*AED*_tot_) values for all investigated herbs, respectively. Lemon Balm (H8) and Lavender (H16) herbs have the lowest and highest *AED*_totalr_ among all herb sample values for all investigated herbs, respectively. According to the NSRC and the International Atomic Energy Agency (IAEA), the annual effective dose equivalent for all tested herbs is less than the annual dose limit of 1 mSv for the general population.

**Figure 8:**
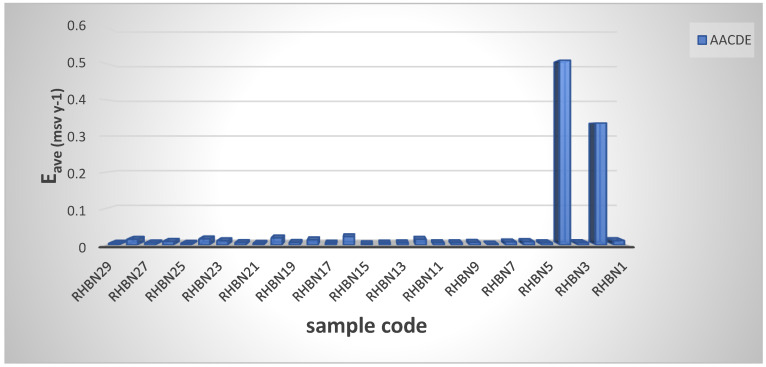
The average annual committed effective dose (Eave) distribution in the various species of the medicinal plant samples.

Table 4 and Figure 8 represent the minimum, maximum, and mean annual effective doses (AACDE) values due to the intake of ^238^U, ^232^Th, and ^40^K radionuclides through eating the medical plants (herbs), which were equal to 0.002304 ± 0.000091, 0.50869 ± 0.00024, and 0.0373 ± 0.00040 mSv/yr, respectively. Lemon Balm (H8) and Worm Wood (H24) herbs have the lowest and highest AACDE among all herb samples. The AACDE values were lower than the global average (0.3 mSv/yr) for natural radionuclide ingestion reported in the UNSCEAR 2000 report [32]. Table 5 compares our AACDE to those assessed in Egypt [55], South India [46], Ghana [2], Iraq [47] and Thailand [56]. According to the comparison, our result is smaller than that of Egypt [55], and the amount of AACDE is higher than that South India [46], Ghana [2], Iraq [47], Turkey [57] and Thailand [56]. These figures are all considerably lower than the global average dose [32]. As a result, the medicinal plant samples tested here are radiologically safe for adult consumption and pose no risk to human health. According to the findings, there are no radiological health concerns associated with the use of these materials.

**Figure 9:**
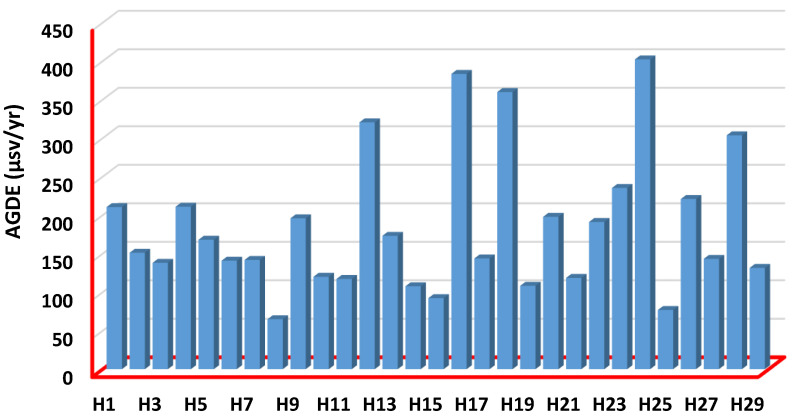
The annual gonadal equivalent dose (AGDA) in the various species of the medicinal plant samples.

The annual gonadal dose equivalent (*AGDE*) for medicinal plants is shown in Figure 9 and listed in Table 4. *AGDE* values range from 402.2 ± 11.3 to 64.8 ± 2.57 µSv/yr with an average of 185.1 ± 6.48 µSv/yr. All values are less than their corresponding global value of 300 μSv/yr [32], except for Worm Wood (H24). These measurements provide information on the local drugs, in order for these models to be used to formulate guidelines related to radiological health care.

The outdoor absorbed dose rate (*D*_outdoor_) values have been estimated for the medicinal plants’ samples, as shown in Table 6. It was found that the values of the *D*_outdoor_ vary from 55.46 ± 1.59 to 8.87 ± 0.35 nGy/h with a mean value of 22.75 ± 22.75 nGy/h. The lowest value was found in the sample Lemon Balm and the highest value in the Worm Woodsample. The values of the absorbed dose rate for all samples were less than the permissible level of 84 nGy/h; according to UNSCEAR, it has been recommended that the average exposure rate of the population should be within 84 nGy/h, while the indoor absorbed dose rate (*D*_indoor_) values ranged from 103.01 ± 1.98 to 16.52 ± 0.65 nGy/h, with an average value of 48.183 ± 1.71 nGy/h. The lowest value was found in Lemon Balm and the highest in a sample Quince. The values of the absorbed dose rate for all samples were less than the permissible level of 84 nGy h^−1^. According to UNSCEAR, the population’s average exposure rate should be kept below 84 nGy/h.

According to UNSCEAR, the average indoor absorbed dose rate values for all samples are below the permissible level of 59 nGy h^−1^. The external and internal hazard indexes are shown in Table 6, and their maximum values are 0.287 ± 0.013 and 0.3099 ± 0.015, respectively. At the same time, the minimum values were 0.0429 ± 0.0017 and 0.0429 ± 0.0017, respectively. The average values were 0.1322 ± 0.0050 and 0.1448 ± 0.0059. For all types of medicinal plant samples evaluated in this study, the calculated values of extrinsic and intrinsic risk indices were less than one [32]. Therefore, there should be efforts to reduce the annual effective dose to ≤1.5 mSv for the safe use of these plants, because of the calculated radioactivity level index in Table 6. The values ranged from 0.860 ± 0.024 maximum value in Worm wood sample to 0.1376 ± 0.0054 minimum value in the Lemon Balm sample, with an average value of 0.399 ± 0.0142. All values of the calculated radioactivity level index (Iγ) for the samples were checked, and were below the permissible levels [32].

Excess lifetime cancer risk (*ELCR*) values ranged from the maximum value 1.7804 × 10^−3^ ± 0.0008 in Thyme herb to the minimum value 0.00806 × 10^−3^ ± 0.003 in Lemon Balm, with an average value of 0.1307 × 10^−3^ ± 0.00142. Based on the annual exposure limit of (1 mSv) for the general population set by UNSCEAR, ICRP [32,58], the mean value of ELCR is less than the global average of 2.9 × 10^−4^ as shown in Table 7.

## 5. Conclusions

The gamma rays released by natural radionuclides, ^238^U, ^232^Th, and ^40^K, were measured in 29 samples of medicinal herbs commonly used in Egypt. The concentration of naturally occurring radionuclide activity in medicinal plant samples was examined for the first time. The average activity concentrations in the examined medicinal herbs were 7.25 ± 0.52, 7.78 ± 0.633, and 471.4 ± 11.33 Bq/Kg, respectively. NORMs were reported to have mean annual effective doses of 0.267 ± 0.0095 and 0.2363 ± 0.0084 mSv/yr from both external and internal exposure (outdoor annual effective doses, indoor annual effective doses) and ingestion of NORMs in the studied medicinal plants at a concentration of 0.0373 ± 0.00040 mSv/yr. We also determined that the findings must be within the UNSCEAR Committee’s allowed limit. The computed radioactivity level index (I) for the tested samples was below the allowed limit, and the absorbed dose rate was within the global average of 84 nGy/h. Since the projected life-long excess cancer risks are globally recognised, the use of these plant samples poses no radiological health hazards. These findings were compared with their respective reference values and with results from other nations. The comparison revealed that the current study’s radioactivity concentrations and annual effective doses were comparable to previous research in other countries. The levels were likewise within UNSCEAR’s allowed limit. The study’s plant samples had no artificial radioactivity. The radiation level of the plant samples in this investigation does not now constitute a health danger. As a result, a continual environmental monitoring program is required to detect any changes caused by artificial radioactivity produced by a nuclear site. Using these plants in herbal medicines may not be harmful to your health. The baseline data from this research may be used to estimate future radiation threats to human health.

## Figures and Tables

**Table 1:** Physical data of herbs.

Herb	Code	Scientific Name	Sample Part
Sage herb	H1	*Salvia officinalis*	Leaves
Guava paper	H2	*Psidium guajava*	Leaves
Margoram herb	H3	*Origanum majorana* L.	Leaves
Thyme herb	H4	*Thymus vulgaris* L. *(T. vulgaris)*	Leaves
Stevia	H5	*(Stevia rebaudiana Bert., Asteraceae)*	Leaves
Senna	H6	*Cassia italic*	Leaves
Halfa-bar	H7	*Cymbopogon schoenanthus* L.	Leaves
Lemon Balm	H8	*Lamiaceae*	Leaves
Argel	H9	*Solenostemma argel*	Stems
Anise stare	H10	*Illicium anisatum* L.	Seed
Mustard	H11	*Brassica nigra* L.	Seed
Agwain	H12	*Trachyspermum ammi*	Seed
Garden cress	H13	*Lepidium sativum*	Seed
Saussurea costus	H14	*Saussurea lappa*	Root
Flax seed	H15	*Linum usitatissimum*	Seed
Lavender	H16	*Lavandula*	Flower
Myrtle	H17	*Myrtus Communis*	Leaves
Basil	H18	*Ocimum basilicum*	Leaves
Barley	H19	*Hordeum vulgare* L.	Seed
Cinnamon	H20	*Cinnamomum, Cassia*	Bark
Fenugreek	H21	*Trigonella foenumgm*	Seed
White ginger	H22	*Zingiber officinale Roscoe*	Root
Quince	H23	*Cydonia oblongaM*	Root
Worm wood	H24	*Artemisia herba-alba*	Leaves
Rhubard	H25	*Rheum palmatum* L.	Root
Spanish Broom	H26	*Spartium junceum* L.	Seed
Turmeric	H27	*Curcuma longa*	Root
Dill	H28	*Anethum graveolens g*	Seed
Fennel	H29	*Foeniculum Vulgare*	Seed

**Table 2:** Specific activities (Bq/kg) of ^238^U (^226^Ra), ^232^Th, and ^40^K in medicinal plant samples using a γ-spectrometer.

Code of Sample	^238^U (Bq/kg)	^232^Th (Bq/kg)	^40^K (Bq/kg)
H1	4.99 ± 0.27	10.72 ± 0.85	478.5 ± 14.9
H2	9.3 ± 0.67	6.33 ± 0.49	305.8 ± 8.58
H3	1.064 ± 0.07	2.84 ± 0.33	391.5 ± 11.07
H4	20.71 ± 1.52	BDL	467.9 ± 11.2
H5	1.49 ± 0.12	BDL	520.6 ± 10.4
H6	BDL	9.92 ± 0.91	316.5 ± 9.02
H7	12.92 ± 0.49	8.49 ± 0.55	211.7 ± 7.41
H8	BDL	BDL	206.5 ± 8.20
H9	BDL	BDL	623.9 ± 13.6
H10	0.359 ± 0.03	3.83 ± 0.47	327.6 ± 11.5
H11	BDL	4.26 ± 0.69	316.5 ± 11.4
H12	3.89 ± 0.19	10.11 ± 0.89	847.9 ± 15.0
H13	13.48 ± 1.44	BDL	418.8 ± 9.78
H14	BDL	3.017 ± 0.31	302.7 ± 8.59
H15	0.105 ± 0.007	BDL	292.8 ± 7.96
H16	9.43 ± 0.72	22.26 ± 2.05	831.9 ± 19.3
H17	22.13 ± 2.17	0.706 ± 0.11	230.7 ± 7.69
H18	2.8 ± 0.19	3.28 ± 0.37	1074.9 ± 19.9
H19	2.53 ± 0.16	7.008 ± 0.69	226.6 ± 7.21
H20	6.5 ± 0.28	29.35 ± 1.33	175.4 ± 6.87
H21	BDL	BDL	377.5 ± 8.66
H22	18.6 ± 1.55	BDL	425.7 ± 10.8
H23	BDL	7.47 ± 0.65	650.1 ± 15.9
H24	2.55 ± 0.21	5.61 ± 0.64	1181.2 ± 25.5
H25	2 ± 0.10	4 ± 0.36	172 ± 5.85
H26	BDL	4.55 ± 0.41	643.2 ± 13.4
H27	BDL	1.203 ± 0.11	440.1 ± 6.13
H28	2.95 ± 0.17	10.78 ± 0.44	794.5 ± 13.2
H29	BDL	BDL	418.8 ± 9.89
**Maximum**	**20.71 ± 1.52**	**29.35 ± 1.33**	**1181.2 ± 25.5**
**Minimum**	**BDL**	**BDL**	**172 ± 5.85**
**Average**	**7.25 ± 0.54**	**7.78 ± 0.63**	**471.4 ± 11.33**

BDL below detection limit.

**Table 3:** The mean activity concentrations (Bq/Kg) of the natural radioactivity of medicinal plant samples in the present were compared with those from similar investigations performed in other countries.

Country	U-238	Th-232	K-40	Reference
Iraq	4.953 ± 0.37	2.916 ± 0.12	219.134 ± 2.24	[45] Kareem et al., 2016
South India	6.34 ± 0.81	5.05 ± 0.7	1895.24 ± 103.95	[46] Chandrashekara and Somashekarappa, 2016
Iraq	38.12 ± 1.619	12.95 ± 0.896	570.70 ± 31.453	[47] Hamza etal.,2020
Ghana	31.8 ± 2.8	56.2 ± 2.3	839.8 ± 11.9	[2] Tettey-Larbi et al., 2013
Bangladesh	12.65 ± 5.20	7.38 ± 3.45	661.1 ± 202.6	[48] Sultana et al.,2020
Jordan	2.63 ± 0.30	1.44 ± 0.18	593.97 ± 63.47	[49] Okoor et al.,2019
Turkey	4.48	1.83	259.2	[50] Kırıs, 2020
Turkey	BDL	BDL	1150.8 ± 315.2	[51] Turhan et al., 2007
Nigeria	5.79 ± 1.51	4.13 ± 0.55	630.03 ± 52.9	[52] Alade et al., 2020
Nigeria	25.02 ± 3.18	(35.09 ± 0.71	324.18 ± 8.69	[53] Njinga et al., 2015
Serbia	2.82	0.63	984.32	[54] Živkovićetal.,2021
**World**	**33**	**45**	**400**	[32] **UNSCEAR., 2000**
**Present study**	**7.25 ± 0.54**	**7.78 ± 0.633**	**471.4 ± 11.33**	

**Table 4:** The outdoor (*AED_outdoor_*) and indoor (*AED_indoor_*) annual effective doses and total annual effective doses (*AED*_tot_) for different medicinal plant samples.

Code of Sample	*AED_outdoor_* (mSv/yr)	*AED_indoor_* (mSv/yr)	*AED_total_* (mSv/yr)	*AACDE* (Ingestion of NORMs mSv/yr)	*AGDE* (µSv/yr)
H1	0.0362 ± 0.0015	0.268 ± 0.011	0.3044 ± 0.0132	0.0119 ± 0.00067	210.47 ± 9.06
H2	0.0262 ± 0.0012	0.196 ± 0.0090	0.2224 ± 0.0102	0.335 ± 0.00043	151.21 ± 6.79
H3	0.0234 ± 0.0008	0.173 ± 0.0064	0.197 ± 0.0072	0.0060 ± 0.00031	138.09 ± 5.05
H4	0.0363 ± 0.0014	0.2770 ± 0.0112	0.3134 ± 0.012	0.5086 ± 0.00024	210.91 ± 8.21
H5	0.0283 ± 0.0006	0.2110 ± 0.0046	0.2391 ± 0.0052	0.0059 ± 0.00012	168.07 ± 3.63
H6	0.0242 ± 0.0011	0.1777 ± 0.0084	0.2025 ± 0.0096	0.00924 ± 0.00062	140.84 ± 6.66
H7	0.0249 ± 0.0010	0.187 ± 0.0080	0.212 ± 0.0091	0.00829 ± 0.00043	141.88 ± 6.13
H8	0.0108 ± 0.0004	0.0810 ± 0.0032	0.091 ± 0.0036	0.00230 ± 0.000091	64.841 ± 2.57
H9	0.0329 ± 0.0007	0.2448 ± 0.0053	0.277 ± 0.0060	0.00696 ± 0.00015	195.90 ± 4.27
H10	0.0204 ± 0.0009	0.1508 ± 0.0071	0.1712 ± 0.0081	0.005891 ± 0.00040	119.98 ± 5.66
H11	0.0138 ± 0.0011	0.1471 ± 0.0081	0.1610 ± 0.0093	0.00598 ± 0.00052	117.18 ± 6.46
H12	0.0546 ± 0.0015	0.4048 ± 0.0115	0.4595 ± 0.0131	0.01560 ± 0.00069	320.52 ± 9.01
H13	0.0288 ± 0.0013	0.2251 ± 0.0103	0.2540 ± 0.0116	0.00576 ± 0.00022	173.15 ± 7.52
H14	0.0182 ± 0.0006	0.1350 ± 0.0050	0.1533 ± 0.0057	0.005115 ± 0.00027	107.65 ± 3.98
H15	0.0155 ± 0.0004	0.1153 ± 0.0031	0.130 ± 0.0035	0.003276 ± 0.000088	92.263 ± 2.50
H16	0.0662 ± 0.0029	0.4891 ± 0.021	0.555 ± 0.024	0.02286 ± 0.0014	383.40 ± 16.8
H17	0.0252 ± 0.0017	0.1942 ± 0.0133	0.2194 ± 0.0150	0.00477 ± 0.00032	143.77 ± 9.55
H18	0.0607 ± 0.0014	0.4521 ± 0.0106	0.512 ± 0.0121	0.01411 ± 0.00045	359.88 ± 8.40
H19	0.0187 ± 0.0009	0.1381 ± 0.0072	0.156 ± 0.0082	0.006770 ± 0.00049	108.26 ± 5.63
H20	0.035 ± 0.0015	0.2565 ± 0.0111	0.291 ± 0.0126	0.01938 ± 0.00086	197.84 ± 8.55
H21	0.0199 ± 0.0004	0.1670 ± 0.0033	0.186 ± 0.0038	0.00421 ± 0.000096	118.53 ± 2.70
H22	0.0329 ± 0.0014	0.2955 ± 0.0112	0.328 ± 0.0126	0.006257 ± 0.00024	191.14 ± 8.18
H23	0.0399 ± 0.0013	0.5053 ± 0.0097	0.545 ± 0.0110	0.01155 ± 0.00055	235.35 ± 7.70
H24	0.0680 ± 0.0019	0.0981 ± 0.0144	0.166 ± 0.0163	0.01662 ± 0.00067	402.22 ± 11.3
H25	0.0132 ± 0.0006	0.2769 ± 0.0046	0.290 ± 0.0053	0.00438 ± 0.00028	76.90 ± 3.63
H26	0.0374 ± 0.0010	0.2589 ± 0.0074	0.296 ± 0.0084	0.00979 ± 0.00038	220.98 ± 5.92
H27	0.0241 ± 0.0004	0.1792 ± 0.0029	0.203 ± 0.0033	0.00560 ± 0.00013	143.21 ± 2.37
H28	0.0535 ± 0.0011	0.3832 ± 0.0083	0.436 ± 0.0094	0.01555 ± 0.00041	303.64 ± 6.50
H29	0.0253 ± 0.0005	0.1643 ± 0.0038	0.1896 ± 0.0043	0.00467 ± 0.00010	131.50 ± 3.07
**Maximum**	**0.0680 ± 0.0019**	**0.5053 ± 0.0097**	**0.555 ± 0.024**	**0.50869 ± 0.00024**	**402.22 ± 11.3**
**Minimum**	**0.0108 ± 0.0004**	**0.0810 ± 0.0032**	**0.0919 ± 0.0036**	**0.002304 ± 0.000091**	**64.841 ± 2.57**
**Average**	**0.0315 ± 0.0011**	**0.2363 ± 0.0084**	**0.267 ± 0.0095**	**0.0373 ± 0.00040**	**185.1 ± 6.48**

**Table 5:** Comparison between *AACED* ingestion dose of the present medicial plant samples with that of other countries of the world.

Country	*AACED* (Ingestion)	Reference
South India	0.0075 to 0.1067	[46] Chandrashekara and Somashekarappa., 2016
Ghana	0.0261 to 0.042	[2] Tettey-Larbi et al., 2013
Iraq	0.010399 to 0.002757	[47] Hamza et al., 2020
Thailand	0.0001 to 0.0327	[56] Kranrod et al., 2016
Egyt	0.6 to 2.0	[55] Ahmed et al., 2010
Turkey	0.3 to 9.0 0.3	[57] Parmaksız and Ağuş, 2014
World		[32] UNSCEAR, 2000
Present study	0.50869 to 0.002304	

**Table 6:** Outdoor and indoor absorbed dose rate, internal hazard index (*H*_in_), external hazard index (*H*_ex_), and radioactivity level index (*I_γ_*) for different medicinal plant samples.

Code of Sample	*D_outdoor_* (nGy/h)	*D_indoor_* (nGy/h)	*H*_in_	*H*_ex_	*I_γ_*
H1	29.55 ± 1.29	54.66 ± 2.37	0.1678 ± 0.0078	0.1558 ± 0.0071	0.459 ± 0.020
H2	21.38 ± 0.98	39.98 ± 1.83	0.1382 ± 0.0072	0.1140 ± 0.0054	0.329 ± 0.0150
H3	19.09 ± 0.71	35.42 ± 1.30	0.0981 ± 0.0039	0.0956 ± 0.0037	0.296 ± 0.011
H4	29.67 ± 1.18	56.48 ± 2.29	0.2092 ± 0.0105	0.1532 ± 0.0064	0.450 ± 0.017
H5	23.072 ± 0.50	43.01 ± 0.94	0.1162 ± 0.0028	0.1122 ± 0.0024	0.357 ± 0.007
H6	19.79 ± 0.95	36.23 ± 1.73	0.1041 ± 0.0054	0.105 ± 0.0054	0.3102 ± 0.015
H7	20.35 ± 0.88	38.16 ± 1.64	0.1466 ± 0.0063	0.1129 ± 0.0049	0.3121 ± 0.0013
H8	8.879 ± 0.35	16.52 ± 0.65	0.0429 ± 0.0017	0.0429 ± 0.0017	0.1376 ± 0.0054
H9	26.82 ± 0.58	49.91 ± 1.08	0.1297 ± 0.0028	0.1297 ± 0.0028	0.415 ± 0.009
H10	16.641 ± 0.80	30.75 ± 1.46	0.0848 ± 0.0043	0.0844 ± 0.0042	0.259 ± 0.012
H11	11.303 ± 0.92	30.00 ± 1.67	0.0582 ± 0.0050	0.0588 ± 0.0050	0.176 ± 0.014
H12	44.55 ± 1.28	82.53 ± 2.35	0.2419 ± 0.0075	0.2272 ± 0.0070	0.692 ± 0.020
H13	23.55 ± 1.08	45.90 ± 2.10	0.1599 ± 0.0098	0.1235 ± 0.0059	0.369 ± 0.016
H14	14.89 ± 0.56	27.53 ± 1.02	0.0745 ± 0.0029	0.075 ± 0.0029	0.231 ± 0.008
H15	12.641 ± 0.34	23.52 ± 0.63	0.0614 ± 0.0016	0.0611 ± 0.0016	0.195 ± 0.0053
H16	53.99 ± 2.43	99.71 ± 4.46	0.309 ± 0.015	0.2874 ± 0.013	0.840 ± 0.038
H17	20.563 ± 1.39	39.59 ± 2.72	0.170 ± 0.0137	0.1105 ± 0.0078	0.308 ± 0.020
H18	49.560 ± 1.17	92.17 ± 2.18	0.251 ± 0.0066	0.244 ± 0.0060	0.768 ± 0.018
H19	15.27 ± 0.81	28.16 ± 1.48	0.087 ± 0.0050	0.0819 ± 0.0045	0.238 ± 0.012
H20	28.83 ± 1.25	52.29 ± 2.26	0.184 ± 0.0080	0.1714 ± 0.0073	0.453 ± 0.019
H21	16.23 ± 0.36	34.05 ± 0.68	0.0784 ± 0.0017	0.0784 ± 0.0017	0.251 ± 0.005
H22	26.88 ± 1.17	60.24 ± 2.29	0.1890 ± 0.0106	0.1387 ± 0.0064	0.407 ± 0.017
H23	32.60 ± 1.08	103.01 ± 1.98	0.1639 ± 0.0058	0.1650 ± 0.0058	0.508 ± 0.017
H24	55.46 ± 1.59	20 ± 2.93	0.2810 ± 0.0089	0.274 ± 0.0083	0.860 ± 0.024
H25	10.81 ± 0.51	56.461 ± 0.95	0.0620 ± 0.0031	0.057 ± 0.0028	0.168 ± 0.008
H26	30.49 ± 0.83	52.77 ± 1.52	0.151 ± 0.0043	0.151 ± 0.0043	0.474 ± 0.013
H27	19.67 ± 0.33	36.53 ± 0.60	0.096 ± 0.0016	0.096 ± 0.0016	0.305 ± 0.0051
H28	43.62 ± 0.92	78.13 ± 1.69	0.238 ± 0.0053	0.224 ± 0.0049	0.677 ± 0.014
H29	20.64 ± 0.42	33.50 ± 0.78	0.099 ± 0.0020	0.099 ± 0.0020	0.320 ± 0.0065
**Maximum**	**55.46 ± 1.59**	**103.01 ± 1.98**	**0.3099 ± 0.015**	**0.2874 ± 0.013**	**0.860 ± 0.024**
**Minimum**	**8.879 ± 0.35**	**16.52 ± 0.65**	**0.0429 ± 0.0017**	**0.0429 ± 0.0017**	**0.137 ± 0.0054**
**Average**	**22.75 ± 0.92**	**48.183 ± 1.71**	**0.1448 ± 0.0059**	**0.1322 ±** **0.0050**	**0.399 ± 0.0142**

**Table 7:** The excess lifetime cancer risk (*ELCR*) for the investigated samples.

Sample	*ELCR* × 10^−3^	Sample	*ELCR* × 10^−3^
H1	0.0417 ± 0.0023	H16	0.0800 ± 0.0050
H2	1.1751 ± 0.0015	H17	0.0167 ± 0.0011
H3	0.0213 ± 0.0011	H18	0.0493 ± 0.0015
H4	1.7804 ± 0.0008	H19	0.0236 ± 0.0017
H5	0.0207 ± 0.0004	H20	0.0678 ± 0.0030
H6	0.0323 ± 0.0022	H21	0.0147 ± 0.0003
H7	0.0290 ± 0.0015	H22	0.0219 ± 0.0008
H8	0.0080 ± 0.0003	H23	0.0404 ± 0.0019
H9	0.0243 ± 0.0005	H24	0.0581 ± 0.0023
H10	0.0206 ± 0.0014	H25	0.0153 ± 0.0009
H11	0.0209 ± 0.0018	H26	0.0342 ± 0.0013
H12	0.0546 ± 0.0024	H27	0.0196± 0.0004
H13	0.0201 ± 0.0007	H28	0.0544 ± 0.0014
H14	0.0179 ± 0.0009	H29	0.0163 ± 0.0003
H15	0.0114 ± 0.0003		
**Maximum**	**1.7804 ± 0.0008**		
**Minimum**	**0.00806 ± 0.003**		
**Average**	**0.1307 ± 0.00142**		

## Data Availability

The data presented in this study are available on request from the corresponding author. All data generated or analysed during this study are included in this published article. Correspondence and requests for materials should be addressed to H.M.Z.

## Abbreviations

**Abbreviation**	**Description**
HPGe	High Purity Germanium
NORM	Naturally Occurring Radioactive Material
IAEA	International Atomic Energy Agency
WHO	World Health Organization
ICRP	International Committee on Radiation Protection
UNSCEAR	United National Scientific Committee on the Effects of Atomic Radiation
NaI	Sodium Iodide
FWHM	Full width at half maximum
B.D.L	Below Detection Limit
AEDE out	Annual effective dose equivalent in the outdoor
AEDE in	Annual effective dose equivalent in the indoor
AEDE (total)	Total Annual effective dose equivalen
Bq/kg	Becquerel per kilogram
*AACED*	annual committed effective doses
*ELCR*	excess lifetime cancer risk
*R*aeq	Radium equivalent
*AGDE*	annual gonadal dose equivalent
*H*ex	external hazard index
NaI(Tl)	Scintillation detector
CLERMIT	central laboratory for Environmental Radioactivity Measurements, Inter-comparison, and Training
NRRA	Nuclear & Radiological Regulatory Authority
*D*_ou*t*_	a bsorbaed gamma dose *out door*
*D*_in_	a bsorbaed gamma dose *In door*
*OECD*	Organisation for Economic Co-operation and Development
*H*in	Internal Hazard Index
A	Activit concentration
SD	standard deviation
ICRP	International Commission on Radiological Protection
*I_γ_*	Gamma activity index
